# Assessing the Application of Large Language Models in Generating Dermatologic Patient Education Materials According to Reading Level: Qualitative Study

**DOI:** 10.2196/55898

**Published:** 2024-05-16

**Authors:** Raphaella Lambert, Zi-Yi Choo, Kelsey Gradwohl, Liesl Schroedl, Arlene Ruiz De Luzuriaga

**Affiliations:** 1 Pritzker School of Medicine University of Chicago Chicago, IL United States; 2 Section of Dermatology University of Chicago Medical Center Chicago, IL United States

**Keywords:** artificial intelligence, large language models, large language model, LLM, LLMs, machine learning, natural language processing, deep learning, ChatGPT, health literacy, health knowledge, health information, patient education, dermatology, dermatologist, dermatologists, derm, dermatology resident, dermatology residents, dermatologic patient education material, dermatologic patient education materials, patient education material, patient education materials, education material, education materials

## Abstract

**Background:**

Dermatologic patient education materials (PEMs) are often written above the national average seventh- to eighth-grade reading level. ChatGPT-3.5, GPT-4, DermGPT, and DocsGPT are large language models (LLMs) that are responsive to user prompts. Our project assesses their use in generating dermatologic PEMs at specified reading levels.

**Objective:**

This study aims to assess the ability of select LLMs to generate PEMs for common and rare dermatologic conditions at unspecified and specified reading levels. Further, the study aims to assess the preservation of meaning across such LLM-generated PEMs, as assessed by dermatology resident trainees.

**Methods:**

The Flesch-Kincaid reading level (FKRL) of current American Academy of Dermatology PEMs was evaluated for 4 common (atopic dermatitis, acne vulgaris, psoriasis, and herpes zoster) and 4 rare (epidermolysis bullosa, bullous pemphigoid, lamellar ichthyosis, and lichen planus) dermatologic conditions. We prompted ChatGPT-3.5, GPT-4, DermGPT, and DocsGPT to “Create a patient education handout about [condition] at a [FKRL]” to iteratively generate 10 PEMs per condition at unspecified fifth- and seventh-grade FKRLs, evaluated with Microsoft Word readability statistics. The preservation of meaning across LLMs was assessed by 2 dermatology resident trainees.

**Results:**

The current American Academy of Dermatology PEMs had an average (SD) FKRL of 9.35 (1.26) and 9.50 (2.3) for common and rare diseases, respectively. For common diseases, the FKRLs of LLM-produced PEMs ranged between 9.8 and 11.21 (unspecified prompt), between 4.22 and 7.43 (fifth-grade prompt), and between 5.98 and 7.28 (seventh-grade prompt). For rare diseases, the FKRLs of LLM-produced PEMs ranged between 9.85 and 11.45 (unspecified prompt), between 4.22 and 7.43 (fifth-grade prompt), and between 5.98 and 7.28 (seventh-grade prompt). At the fifth-grade reading level, GPT-4 was better at producing PEMs for both common and rare conditions than ChatGPT-3.5 (*P*=.001 and *P*=.01, respectively), DermGPT (*P*<.001 and *P*=.03, respectively), and DocsGPT (*P*<.001 and *P*=.02, respectively). At the seventh-grade reading level, no significant difference was found between ChatGPT-3.5, GPT-4, DocsGPT, or DermGPT in producing PEMs for common conditions (all *P*>.05); however, for rare conditions, ChatGPT-3.5 and DocsGPT outperformed GPT-4 (*P*=.003 and *P*<.001, respectively). The preservation of meaning analysis revealed that for common conditions, DermGPT ranked the highest for overall ease of reading, patient understandability, and accuracy (14.75/15, 98%); for rare conditions, handouts generated by GPT-4 ranked the highest (14.5/15, 97%).

**Conclusions:**

GPT-4 appeared to outperform ChatGPT-3.5, DocsGPT, and DermGPT at the fifth-grade FKRL for both common and rare conditions, although both ChatGPT-3.5 and DocsGPT performed better than GPT-4 at the seventh-grade FKRL for rare conditions. LLM-produced PEMs may reliably meet seventh-grade FKRLs for select common and rare dermatologic conditions and are easy to read, understandable for patients, and mostly accurate. LLMs may play a role in enhancing health literacy and disseminating accessible, understandable PEMs in dermatology.

## Introduction

Health literacy has been well-explored to be a predictor of health outcomes. Differences in health literacy levels have been associated with increased hospitalization and emergency care use, as well as decreased mammography, vaccinations, and medication compliance. Importantly, health literacy has been shown to be implicated in widening existing disparities [1]. However, improving written materials can increase health knowledge, especially when used in combination with brief in-office counseling [2].

Medical professionals play a key role in developing and distributing accurate, readable, and comprehensible medical information to patients across different communities. The current reading level in the United States is rated at a seventh- to eighth-grade level, with the latest assessment results available through the Program for the International Assessment of Adult Competencies for each US state and county. However, because up to 20% of individuals read below the fifth-grade level, the Agency for Healthcare Research and Quality (AHRQ) recommends producing written health care materials at a fourth- to sixth-grade level to maximize readability [3]. Readability in the United States is most commonly assessed with the Flesch-Kincaid reading level (FKRL), a formula that approximates the reading grade level of a given text taking into account sentence, word, and syllable counts [4].

Within dermatology, an evaluation of 706 patient-oriented materials of dermatology was shown to be written at a mean 12th-grade reading level [5]. Further, previous analysis of dermatologic patient education materials (PEMs) available through the American Academy of Dermatology (AAD), WebMD, and Wikipedia had average FKRLs of 9.6, 9.3, and 11.8, respectively [6]. When looking at specific dermatologic diseases, there are studies regarding patient-oriented materials of acne keloidalis nuchae, pemphigus vulgaris, bullous pemphigoid, and epidermolysis bullosa, which showed that most handouts are difficult to read and have a reading level above an eighth-grade level [7-9]. Similar results have been seen with the assessment of dermatologic materials written in Spanish [10]. As such, the average patient may struggle to sufficiently understand and process the dermatologic information available on the web or in the office.

ChatGPT is a large language model (LLM) that uses deep learning algorithms trained on vast amounts of data to generate humanlike responses to user prompts [11]. It is currently being explored as a tool across professions including medicine. When challenged, it performed above the passing score on the National Board of Medical Examiners-Free-Step-1 data set and the United Kingdom Dermatology Specialty Certificate Examination [12]. It has also performed satisfactorily in answering physician-generated medical queries across 12 distinct specialties, including ophthalmology, dermatology, oncology, infectious disease, neurosurgery, gastroenterology, radiation oncology, trauma surgery, cardiology, anesthesiology, pulmonology, and surgical oncology [9]. Since the mainstream introduction of ChatGPT in fall 2022, additional natural language processing models such as GPT-4, DocsGPT (a Doximity and OpenAI collaboration), and the dermatology-specific DermGPT have also been made available, although research on their performance and applications remains lacking [13,14]. While ChatGPT has been shown to appropriately answer patient queries in dermatology, generated answers have not yet been assessed for patient readability [15]. Given their functionality, LLMs have the potential to be a tool to help the clinician workflow and improve patient care [16]. Regarding health literacy, LLMs could be applied to generating PEMs at a specified reading level. When prompted, LLMs attempt to generate documents according to the specifications given. However, whether the generated documents meet the specifications requested must be verified. In this way, the application of LLMs as tools for generating patient handouts at specific reading levels has yet to be explored. Additionally, with the choice between numerous LLMs, it is essential to objectively evaluate the functionality of each.

Here, we assess the application of ChatGPT-3.5, GPT-4, DocsGPT, and DermGPT in generating dermatologic PEMs at specified reading levels at or below the average US adult reading level for both common and rare dermatologic conditions. In addition to assessing the readability of each PEM, we also assess the preservation of meaning between LLM-generated PEMs and AAD PEMs for a given condition. This work may inform future clinician workflows both within and outside of dermatology and allow clinics to efficiently create PEMs that are readable and comprehensible to all patient populations.

## Methods

### Ethical Considerations

No ethics board review was sought as this project does not involve human participants or ethically sensitive materials.

### Study Design

The FKRL of current AAD PEMs was evaluated using Microsoft Word (Microsoft Corp) readability statistics for 4 common (atopic dermatitis [AD], acne vulgaris, psoriasis, and herpes zoster) and 4 rare (epidermolysis bullosa, lichen planus, bullous pemphigoid, and lamellar ichthyosis) dermatologic conditions. Next, ChatGPT-3.5, GPT-4, DermGPT, and DocsGPT were independently prompted to “Create a patient education handout about [common or rare condition] at a [FKRL]” to iteratively generate 10 PEMs per condition at unspecified fifth- and seventh-grade FKRLs. The same prompt was used for each iteration across each LLM. The FKRL of the LLM-generated PEMs was also evaluated using Microsoft Word readability statistics. The preservation of meaning across LLM-generated PEMs was assessed by 2 blinded dermatology resident trainees (LS and KG) using a standardized scoring rubric that assessed a copy of each LLM-generated document at unspecified FKRLs for both common and rare diseases for ease of reading, understandability for patients, and overall accuracy (5 points per domain for an overall total of 15 possible points; Multimedia Appendix 1). Rubrics also provided space for free-response comments. Additionally, members of the University of Chicago Health Literacy Department reviewed representative AAD PEMs and LLM-produced PEMs to provide qualitative feedback on the readability of such documents in line with their plain language guidelines (Multimedia Appendix 2).

### Statistical Analysis

Simple descriptive statistics were performed using Microsoft Excel (Microsoft Corp) and RStudio (Posit PBC). Fisher exact tests were performed in RStudio (Posit) at the *P*=.05 significance level.

## Results

In total, 960 PEMs were generated across 4 LLMs and 8 dermatologic conditions. The average FKRL for each common and rare condition across each LLM and prompt category is shown in Table 1. ChatGPT-3.5 created materials at or below the specified fifth- or seventh-grade FKRL in 53% (43/80) and 65% (52/80) of iterations, respectively; GPT-4 created materials at or below the fifth- or seventh-grade FKRL in 86% (69/80) and 45% (36/80) of iterations, respectively; DocsGPT created materials at or below the specified fifth- or seventh-grade FKRL in 48% (38/80) and 75% (60/80) of iterations, respectively; and DermGPT created materials at or below the specified fifth- or seventh-grade FKRL in 5% (4/80) and 40% (32/80) of iterations, respectively (Tables 2-4).

When prompted to generate PEMs at a fifth-grade reading level, there were no significant differences between DocsGPT and ChatGPT-3.5; both LLMs were able to generate appropriate handouts for common and rare conditions (*P*=.92). However, when compared to DermGPT, both DocsGPT (*P*<.001) and ChatGPT-3.5 (*P*<.001) were better able to generate PEMs at a fifth-grade reading level for common and rare conditions, respectively. When prompted to generate PEMs at a seventh-grade reading level, DocsGPT was better than DermGPT for common conditions (*P*=.04).

Finally, we compared the individual LLM’s ability to generate PEMs about common and rare conditions at either a fifth-grade reading level or a seventh-grade reading level. No difference was observed in the ability of ChatGPT-3.5 or GPT-4 to create PEMs meeting either a fifth-grade or seventh-grade reading level for both common and rare conditions (*P*<.001). DocsGPT, however, was better at creating PEMs meeting a seventh-grade than fifth-grade reading level for both common (*P*=.01) and rare (*P*=.03) conditions. Likewise, DermGPT was better at creating PEMs meeting a seventh-grade than fifth-grade reading level for both common (*P*<.001) and rare (*P*<.001) conditions.

Results from the preservation of meaning analysis revealed that for common conditions, handouts generated by DermGPT ranked the highest for overall ease of reading, patient understandability, and accuracy (14.75/15, 98%), followed by DocsGPT (14.25/15, 95%), ChatGPT-3.5 (13.5/15, 90%), and GPT-4 (13/15, 87%). For rare conditions, handouts generated by GPT-4 ranked the highest (14/15, 93%), followed by ChatGPT-3.5 (13.5/15, 90%), DermGPT (13/15, 87%), and DocsGPT (13/15, 87%). Resident reviewers commented on several key issues present throughout the LLM-generated PEMs. References were often included in PEMs that were left blank or not in alignment with the main purpose of the PEM (eg, a psoriasis PEM citing acne literature). Some references cited by LLMs were also found to be untraceable after a thorough literature search.

Qualitative analysis of AAD PEMs and select LLM-generated PEMs by the University of Chicago Urban Health Initiative Office of Diversity, Equity, and Inclusion’s Health Literacy team was notable for the frequent use of multisyllable, “high-literacy” words across PEMs. Such words, including “permanently,” “whether,” and “environment,” may be difficult for the average reader to understand. Further, individual sentences and paragraphs were often found to be too long for the average reader. Most documents’ content was found to require prior medical knowledge to sufficiently comprehend, as many medical terms were frequently not defined within the handout. Formatting issues, including headings posed as questions and inconsistent bullet-point use, were other commonly encountered issues in both AAD and LLM-produced PEMs that may further limit their readability.

**Table 1:** Average Flesch-Kincaid reading levels (FKRLs) for patient education handouts generated by ChatGPT-3.5, GPT-4, DocsGPT, and DermGPT.

FKRLs		AAD^a,b^	ChatGPT-3.5, mean (SD)				GPT-4, mean (SD)				DocsGPT, mean (SD)				DermGPT, mean (SD)		
			Not specified^c^	Fifth grade^d^	Seventh grade^e^	Not specified		Fifth grade	Seventh grade	Not specified		Fifth grade	Seventh grade	Not specified		Fifth grade	Seventh grade
**Common conditions**																	
	Acne vulgaris	8.5	11.77 (0.13)	5.13 (0.43)	5.99 (0.85)	9.95 (0.98)		3.91 (0.43)	5.65 (1.03)	10.0 (1.02)		3.76 (0.51)	4.56 (0.26)	9.23 (0.5)		7.22 (0.46)	7.19 (0.34)
	Atopic dermatitis	9.1	11.73 (0.13)	4.94 (0.68)	7.25 (0.17)	10.19 (0.56)		4.26 (0.33)	7.03 (0.83)	10.06 (0.96)		5.78 (0.47)	7.2 (0.88)	12.74 (0.19)		6.9 (0.95)	6.6 (0)
	Herpes zoster	8.6	9.59 (0.14)	5.47 (0.63)	6.3 (1.1)	9.12 (0.88)		3.65 (0.25)	6.94 (0.68)	10.01 (0.68)		4.96 (0.28)	5.28 (0.49)	11.38 (0.93)		8.98 (0.17)	8.9 (0)
	Psoriasis	11.2	11.75 (0.32)	4.55 (1.15)	6.71 (0.95)	9.92 (0.57)		5.06 (0.2)	8.05 (0.76)	10.63 (0.89)		5.68 (0.43)	6.87 (1.08)	11.2 (0.86)		6.63 (1.36)	6.68 (0.62)
	Average FKRL across common conditions	9.35 (1.26)^f^	11.21 (1.08)	5.02 (0.38)	6.56 (0.55)	9.795 (0.47)		4.22 (0.61)	6.92 (0.98)	10.18 (0.3)		5.01 (0.93)	5.98 (1.26)	11.14 (1.45)		7.43 (1.06)	7.27 (1.15)
**Rare conditions**																	
	Bullous pemphigoid	8.4	12.09 (0.19)	4.57 (1.14)	6.91 (1.06)	9.65 (0.77)		4.24 (0.29)	7.37 (0.53)	9.98 (0.52)		6.11 (0.47)	6.86 (1.09)	11.67 (0.05)		7.34 (0.92)	9.39 (1.2)
	Epidermolysis bullosa	12.3	11.36 (0.23)	5.54 (0.79)	7.62 (0.88)	11.32 (0.65)		5.42 (0.51)	9.62 (0.94)	10.8 (0.44)		4.55 (0.4)	6.1 (0.7)	13.77 (0.09)		8.68 (0.82)	8.54 (0.13)
	Lamellar ichthyosis	10.3	11.63 (0.34)	5.19 (1.0)	5.92 (0.98)	9.51 (0.57)		4.08 (0.27)	5.77 (1.27)	11.08 (0.61)		5.66 (0.72)	6.75 (0.91)	11.68 (0.55)		6.6 (0)	6.6 (0)
	Lichen planus	7	10.73 (0.52)	5.21 (0.35)	6.53 (0.51)	8.92 (0.27)		4.08 (0.37)	7.06 (0.79)	9.77 (0.78)		4.88 (0.23)	6.01 (0.93)	10.6 (0.32)		5.95 (0.2)	5.77 (0.13)
	Average FKRL across common conditions	9.50 (2.3)^f^	11.45 (0.57)	5.13 (0.4)	6.75 (0.71)	9.85 (1.03)		4.46 (0.65)	7.46 (1.6)	10.41 (0.63)		5.30 (0.71)	6.43 (0.44)	11.93 (1.33)		7.14 (1.17)	7.58 (1.68)

^a^AAD: American Academy of Dermatology.

^b^Values are expressed as handouts per disease or condition.

^c^When prompted to create patient education handouts without specifying reading level.

^d^When prompted to create patient education handouts at a fifth-grade reading level.

^e^When prompted to create patient education handouts at a seventh-grade reading level.

^f^Values are expressed in mean (SD).

**Table 2:** Handouts generated by ChatGPT-3.5, GPT-4, DocsGPT, and DermGPT that meet the prompted reading level.

Handouts generated at or below the specified reading level			ChatGPT-3.5, n (%)			GPT-4, n (%)					DocsGPT, n (%)					DermGPT, n (%)		
			Fifth-grade reading level^a^	Seventh-grade reading level^b^		Fifth-grade reading level		Seventh-grade reading level		Fifth-grade reading level			Seventh-grade reading level		Fifth-grade reading level			Seventh-grade reading level
**Common conditions**																		
	Acne vulgaris (n=10)	6 (60)		9 (90)	10 (100)		9 (90)		10 (100)			10 (100)		0 (0)			3 (30)	
	Atopic dermatitis (n=10)	7 (70)		0 (0)	10 (100)		5 (50)		1 (10)			3 (30)		0 (0)			10 (100)	
	Herpes zoster (n=10)	1 (10)		8 (80)	10 (100)		7 (70)		6 (60)			10 (100)		0 (0)			0 (0)	
	Psoriasis (n=10)	9 (90)		7 (70)	6 (60)		1 (10)		0 (0)			6 (60)		0 (0)			6 (60)	
	Total (n=40)	23 (57)		24 (60)	36 (90)		22 (55)		17 (42)			29 (72)		0 (0)			19 (47)	
**Rare conditions**																		
	Bullous pemphigoid (n=10)	9 (90)		9 (90)	10 (100)		2 (20)		0 (0)			5 (50)		0 (0)			2 (20)	
	Epidermolysis bullosa (n=10)	1(10)		1 (10)	3 (30)		0 (0)		9 (90)			10 (100)		0 (0)			0 (0)	
	Lamellar ichthyosis (n=10)	8 (80)		9 (90)	10 (100)		9 (90)		4 (40)			7 (70)		0 (0)			10 (100)	
	Lichen planus (n=10)	2 (20)		9 (90)	10 (100)		3 (30)		8 (80)			9 (90)		4 (40)			10 (100)	
	Total (n=40)	20 (50)		28 (70)	33 (82)		14 (35)		21 (52)			31 (77)		4 (10)			22 (55)	

^a^When prompted to create patient education handouts at a fifth-grade reading level.

^b^When prompted to create patient education handouts at a seventh-grade reading level.

**Table 3:** LLM^a^-generated handouts meeting a prompted fifth- or seventh-grade reading level for common dermatoses.

LLM	Handouts meeting prompted fifth-grade reading level (n=40), n (%)	Handouts meeting prompted seventh-grade reading level (n=40), n (%)
ChatGPT-3.5	23 (58)	24 (60)
GPT-4	36 (90)	22 (55)
DocsGPT	17 (43)	29 (73)
DermGPT	0 (0)	19 (48)

^a^LLM: large language model.

**Table 4:** LLM^a^-generated handouts meeting a prompted fifth- or seventh-grade reading level for rare dermatoses.

LLM	Handouts meeting prompted fifth-grade reading level (n=40), n (%)	Handouts meeting prompted seventh-grade reading level (n=40), n (%)
ChatGPT-3.5	20 (50)	28 (70)
GPT-4	33 (83)	14 (35)
DocsGPT	21 (53)	32 (78)
DermGPT	4 (10)	22 (55)

^a^LLM: large language model.

## Discussion

### Principal Findings

Studies on interventions to improve care for patients with limited health literacy show that it is important to [17] improve patient-centered communication, use clear communication techniques, reinforce teaching with confirmation of understanding, use visual aids, use clear medication labeling, develop clear health education materials, and use specialized health educators.

Patient education initiatives have been shown to be effective in dermatology, particularly for common dermatologic conditions such as AD and acne vulgaris. Specific to AD, patient educational initiatives implemented to improve the management of AD have resulted in a significant improvement in severity and quality of life for pediatric and adult patients [18-20]. Similarly, for patients with acne vulgaris, those who received audiovisual education materials regarding their condition showed significant improvements of their acne as well as increased treatment adherence and overall patient satisfaction [21,22]. One study focusing on written eczema action plans for parents whose children have AD showed improvements in child eczema based on this intervention [23]. Despite these successes, educational initiatives and interventions can be time-consuming and challenging to incorporate to a clinic workflow.

Few initiatives have focused on improving the readability of dermatologic PEMs that can easily be distributed at the end of a clinic visit. Studies demonstrate the association of low health literacy with worsened health outcomes and the success of educational interventions on patient outcomes [1,2]. As such, tools that help clinics create patient handouts at an appropriate US reading level (seventh- to eighth-grade level) may be an important factor in patient outcomes.

Larger academic institutions such as the University of Chicago have ancillary support through the Urban Health Initiative Office of Diversity, Equity, and Inclusion that offers services to review and edit existing patient handouts to meet health literacy standards. These standards strictly follow the Patient Education Materials Assessment Tool prepared by the AHRQ of the US Department of Health and Human Services [24]. Unlike standard readability software, human assessment of readability allows for a more nuanced, qualitative review that may be better able to assess how sentence structure, document formatting, and the inclusion of figures or images impact readability. However, these resources are not widely available and require considerable human effort, leaving smaller groups and independent practices largely unsupported. Further, such review may be subject to human error or bias, particularly if standardized rubrics or guidelines are not available.

This work is the first to assess the application of LLMs in generating dermatologic PEMs at specified reading levels. Our analysis suggests that LLM-produced PEMs may reliably meet seventh-grade FKRLs for select common and rare dermatologic conditions and are easy to read, understandable for patients, and mostly accurate. More specifically, GPT-4 appeared to outperform ChatGPT-3.5, DocsGPT, and DermGPT at the fifth-grade FKRL, although both ChatGPT-3.5 and DocsGPT performed better at the seventh-grade FKRL for rare conditions. Although the seventh-grade reading level is slightly outside that recommended by AHRQ for PEMs (fourth- to sixth-grade FKRL), LLMs consistently produced PEMs at lower reading levels compared to currently available AAD PEMs for the same conditions. As such, LLMs may play a role in enhancing health literacy and disseminating accessible, understandable PEMs in dermatology. Importantly, if using LLMs to create PEMs, this study demonstrates the importance of specifying an FKRL in the prompt. Without specification, all LLMs consistently generate handouts above the average US reading level.

### Limitations

Key limitations of this work include the limited number of iterations per LLM prompt (n=10) as well as the limited number of common (n=4) and rare (n=4) diseases selected to study. Further, reliability assessment may be subject to reviewer bias and is limited by a small sample (n=2) of reviewers. The ability of LLMs to appropriately cite sources and produce factual information remains an area of continued improvement. Recently, novel LLMs using retrieval-augmented capabilities have been designed specifically for clinical practice to help enhance the ability of LLMs to produce factual, clinically relevant information [25]. However, the ability of these newer LLMs to sound human has limited their use [25]. Further, LLMs may benefit from prompt optimization techniques to produce the best outputs, which may require more time and effort than is feasible for clinician users [26]. Together, these issues may hinder the ability of LLMs to produce ready-to-share PEMs, which may result in extra time spent by clinical staff in fact-checking or formatting materials for dissemination. Some platforms, including GPT-4, DocsGPT, and DermGPT, require memberships or paid subscriptions or may have waitlists, which may limit their accessibility. The accuracy and readability of LLM-generated PEMs in multiple languages may present additional hurdles and warrant further investigation. Further, building trust by patients and providers in materials generated by LLMs remains to be explored. Ethical dilemmas surrounding the use of LLMs in dermatology must also consider whether the benefit of more accessible dermatologic information outweighs the risks of sharing potentially inaccurate or incomplete information [27,28]. To this effect, recent literature demonstrates that ChatGPT-3.5’s responses to queries about common dermatologic skin conditions may be lacking in both accuracy and comprehensiveness [15]. As such, it is important to emphasize the use of LLMs in producing PEMs as a tool and not as a replacement to physician-written PEMs.

### Conclusions

LLMs such as ChatGPT-3.5, GPT-4, DocsGPT, and DermGPT may be useful in generating dermatology PEMs for select common and rare diseases at the seventh-grade FKRL. With prompting, LLMs consistently produce PEMs at lower reading levels than AAD PEMs for the same conditions and may be a useful supplementary tool in sharing appropriately readable dermatologic information with patients.

## Appendix

Multimedia Appendix 1 Scoring rubric for preservation of meaning analysis.

Multimedia Appendix 2 Plain language guidelines.

## Abbreviations

AAD: American Academy of Dermatology
AD: atopic dermatitis
AHRQ: Agency for Healthcare Research and Quality
FKRL: Flesch-Kincaid reading level
LLM: large language model
PEM: patient education material
