# Supplementary material for: Assessing the Application of Large Language Models in Generating Dermatologic Patient Education Materials According to Reading Level: Qualitative Study
Source: JMIR Dermatol. 2024 May 16;7:e55898. doi: 10.2196/55898 (PMC11140271; doi:10.2196/55898)
Supplement: Multimedia Appendix 1 [file derma_v7i1e55898_app1.docx]

Document #:

Reviewer:

- Please mark each incorrect fact or statement with a comment (if scoring electronically) or an X (if scoring on a printed copy).
- For any references cited within the handout, please comment briefly on the relevance of the given citation (ex: if an acne handout incorrectly cites a paper about psoriasis, please make note).
- After reading each NLPM generated handout, score each individual handout using the following criteria:
  - This handout is easy to read.
    - 1 = strongly disagree
    - 2 = disagree
    - 3 = neutral
    - 4 = agree
    - 5 = strongly agree
  - My patients in clinic would understand this handout.
    - 1 = strongly disagree
    - 2 = disagree
    - 3 = neutral
    - 4 = agree
    - 5 = strongly agree
  - The information in this handout is accurate.
    - 1 = strongly disagree
    - 2 = disagree
    - 3 = neutral
    - 4 = agree
    - 5 = strongly agree
