# Supplementary material for: Assessing the Application of Large Language Models in Generating Dermatologic Patient Education Materials According to Reading Level: Qualitative Study
Source: JMIR Dermatol. 2024 May 16;7:e55898. doi: 10.2196/55898 (PMC11140271; doi:10.2196/55898)
Supplement: Multimedia Appendix 2 [file derma_v7i1e55898_app2.pdf]

✓ Plain Language Guidelines

Use these tips for creating easy to read and understand patient materials.

Writing Style

	**Focus on key points.** Leave out things not needed to get your point across.
	**Use plain everyday language.** No medical jargon or technical words. Define medical terms in non-medical language. **Example:** You have high blood pressure, also called hypertension.
	**Define new words and acronyms.** Avoid abbreviations.
	**Use short paragraphs with one idea or subject**. Start paragraphs with a clear topic sentence. Have no more than 3-4 sentences in a paragraph.
	**Use short sentences.** Limit sentences to 6-10 words and one single, easy to understand idea. Be simple, short and to the point. Break longer sentences into 2.
	**Use short, specific and concrete words.** Use words with 1-2 syllables that have only one meaning. Avoid words like shall, should or need. Should has a shaming connotation.
	**Avoid redundancies.** Do not repeat the same information or words unnecessarily.
	**Omit unnecessary words and qualifiers.** They add no additional meaning to a sentence.
	**Do not use special characters.** Extra characters and symbols such as contractions, quotation marks and exclamation points can be mistaken for a letter by lower literacy readers.
	**Do not use "and/or".** This makes the meaning of a sentence unclear. Decide what you want to say and choose one. The / is a special character.
	**Avoid statistics.** If you must use statistics, make them easy to understand. Use simple tables that relate to the information. Pie charts are easier to understand than bar graphs.

Font, Lettering and Page Layout

	**Have large, clear headings.** Use at least a 16pt font for heading at the top of a page or for a new section. Make subheadings (such as for a paragraph) at least 14pt font.
	**Do not use all capital letters.** THEY ARE MORE DIFFICULT AND SLOWER TO READ.
	**Use a font large enough to read.** Do not use a font smaller than Arial 12pt for text. Use an easy to read font without curls or marks (sans serif), such as Arial or Gotham Book.
	**Align text on the left side to increase readability.** Do not "justify" or center paragraph text. This decreases readability by slowing eye movement from irregular spacing.
	**Use bullets or a numbered list.** Put list items in a logical order.
	**Space text so it is easy to read.** If you use size 12 font have at least that same size space or larger between paragraphs. Use at least 1.5 spacing between bullet points and numbered list. Use 1.15 spacing between sentences in a paragraph. Do not indent paragraphs.
	**Use culturally relevant pictures and graphics. Do not use cartoons.** Not all audiences understand cartoons or take them seriously. Do not use images without copyright permission.
	**Follow our Branding Guidelines. If resizing a logo, lock the height and width ratio.**
	**Leave lots of empty space without text or visuals on the page.** Aim for 10-35%. Have at least 0.5 for each top, bottom and side margin.

Other	Other Things to Remember
	**Understand that "readability" level formulas are not always reliable.** (Example: Flesch Kinkaid from Microsoft Word) They do not take into consideration things such as prior knowledge needed to understand abstract words versus concrete words. They do not consider that words can have more than one meaning.
	**Check for correct names, titles, phone numbers, emails and web links.**
	**Proofread aloud** to find errors and test for overall flow and clarity. Ask people unfamiliar with the project to read and edit.
	**Cite references** or evidence for document content if taken from another source.
	**Engage stakeholders** early in the planning process

Make Headings Meaningful to Your Audience

Give a statement to introduce the content. Tell why they need to know. Capture their attention so they keep reading.

Chunk the content into sections with bold headings

- Make the information actionable.
- Start the phrase or sentence with a verb if you want them to do something.
- Put items in a logical order.
- Use periods at the end only if it is a full sentence.

Use bullets for listed items

- **Use positive statements.**
  - Positive: Always eat breakfast.
  - Negative: Do not skip breakfast.
- **Use active voice.** The subject does something, rather than something being done to the subject.
  - Active: The manager told Mr. Doe to provide more information.
  - Passive: Mr. Doe was told by the manager to provide more information.
- **Avoid false subjects.** They displace the real subject.
  - False subject: It is possible that the patient may not qualify for the credit.
  - Real subject: The patient may not qualify for the credit.
- **Use personal pronouns.** This helps patients personally relate to documents.
  - Example: Take your medication in the morning and in the evening.
  - Not: Take twice a day.
